# Fcγ Receptor IIB Controls Skin Inflammation in an Active Model of Epidermolysis Bullosa Acquisita

**DOI:** 10.3389/fimmu.2019.03012

**Published:** 2020-01-14

**Authors:** Balint Kovacs, Jenny Tillmann, Lisa-Christin Freund, Falk Nimmerjahn, Christian D. Sadik, Katja Bieber, Ralf J. Ludwig, Christian M. Karsten, Jörg Köhl

**Affiliations:** ^1^Institute for Systemic Inflammation Research, University of Lübeck, Lübeck, Germany; ^2^Department of Dermatology, University of Lübeck, Lübeck, Germany; ^3^Department of Biology, Chair of Genetics, University of Erlangen-Nuremberg, Erlangen, Germany; ^4^Lübeck Institute for Experimental Dermatology, University of Lübeck, Lübeck, Germany; ^5^Division of Immunobiology, Cincinnati Children's Hospital and College of Medicine, University of Cincinnati, Cincinnati, OH, United States

**Keywords:** Fcγ receptor, epidermolysis bullosa acquisita, autoimmunity, neutrophils, IgG, animal model, pemphigoid disease

## Abstract

Epidermolysis bullosa acquisita (EBA) is an autoimmune skin blistering disease characterized by IgG autoantibodies (aAb) against type VII collagen (COL7). The mechanisms controlling the formation of such aAbs and their effector functions in the skin tissue are incompletely understood. Here, we assessed whether the inhibitory IgG Fc receptor, FcγRIIB, controls the development of autoimmune skin blistering disease in an active model of EBA. For this purpose, we immunized congenic EBA-susceptible B6.SJL-H2s (B6.s) and B6.s-*Fcgr2b*^−/−^ mice with the immunodominant vWFA2 region of COL7. B6.s-*Fcgr2b*^−/−^ mice developed a strong clinical phenotype with 15 ± 3.3% of affected body surface area at week 4. In contrast, the body surface area in B6.s mice was affected to a maximum of 5% at week 6 with almost no disease signs at week 4. Surprisingly, we already found strong but similar COL7-specific serum IgG1 and IgG2b aAb production at week 2. Further, aAb and C3b deposition in the skin of B6.s and B6.s-*Fcgr2b*^−/−^ mice increased between weeks 2 and 6 after vWFA2 immunization. Importantly, neutrophil skin infiltration and activation was much stronger in B6s-*Fcgr2b*^−/−^ than in B6.s mice and already present at week 2. Also, the early aAb response in B6.s-*Fcgr2b*^−/−^ mice was more diverse than in wt B6.s mice. Reactive oxygen species (ROS) release from infiltrating neutrophils play a crucial role as mediator of skin inflammation in EBA. In line, sera from B6.s and B6.s-*Fcgr2b*^−/−^ mice induced strong ROS release from bone marrow-neutrophils *in vitro*. In contrast to the antibody-transfer-induced EBA model, individual targeting of FcγRIII or FcγRIV decreased ROS release to 50%. Combined FcγR blocking abrogated ROS release from BM neutrophils. Also, ROS release induced by COL7-specific serum IgG aAbs was significantly higher using BM neutrophils from B6.s-*Fcgr2b*^−/−^ than from B6.s mice. Together, our findings identified FcγRIIB as a suppressor of skin inflammation in the active EBA model through inhibition of early epitope spreading, protection from strong early neutrophil infiltration to and activation of neutrophils in the skin and suppression of FcγRIII activation by IgG1 aAbs which drive strong ROS release from neutrophils leading to tissue destruction at the dermal-epidermal junction.

## Introduction

FcγRs are a heterogeneous receptor family. Aggregation by IgG immune complexes (IC) results in the initiation of activating or inhibitory signaling cascades. With the exception of the inhibitory FcγRIIB, all other FcγRs trigger cellular activation. FcγRs are predominantly expressed by innate immune cells. The relative distribution of activating/inhibitory FcγR expression sets the threshold of innate immune cell activation through IgG ICs (1). FcγRs play an important role in the host defense against pathogens as receptors for pathogen phagocytosis. Further, they promote protective immunity against pathogens (2).

In addition to their important and desirable role in immune defense, FcγRs may also contribute to disease pathogenesis, especially in autoimmune diseases. In this setting, aAbs activate myeloid cells through FcγR aggregation after the formation of soluble or tissue-bound ICs comprising their cognate auto-antigens (3). Therefore, IC/FcγR interactions have become a potential drug target for the treatment of aAb-mediated diseases such as rheumatoid arthritis or pemphigoid diseases. More specifically, IC/FcγR interactions can be blocked by modulating Fc-glycosylation patterns (4, 5) or by competitive binding of soluble FcγRIIB (sCD32, SM101) to IC (6, 7). Hence, understanding FcγR biology has facilitated the drug development in autoantibody-mediated diseases. This is of particular interest for pemphigoid diseases, where high dose corticosteroids, often associated with severe adverse events, form the backbone of the therapeutic regimen (8, 9).

Due to the availability of a highly reproducible animal model (10), the pemphigoid disease EBA has been relatively well-studied in the past (11). In EBA, aAbs directed against COL7, an integral structural protein of the skin, cause chronic subepidermal blistering, a variable degree of cutaneous inflammation and scaring, as well as organ damage at sites of COL7 expression including the gastro-intestinal tract, eyes, and larynx (12).

The generation of the autoimmune response in EBA depends on B cells, dendritic cells and macrophages as antigen-presenting cells, CD4^+^ T helper cells and neutrophils as the dominant effector cells in skin lesions (13). At the molecular level, formation of anti-COL7 humoral immune response is enhanced by GM-CSF (14). The initial step in disease development is the binding of anti-COL7 aAbs to their target antigen, which is predominantly expressed in the skin. The formation of tissue-bound IC in the skin drives the activation of the complement system, a ß2-integrin-mediated migration of myeloid cells to skin and subsequent production and release of proinflammatory mediators including cytokines, chemokines and leukotrienes (15–18). Within the skin, myeloid cells interact with the ICs. In humans, this process depends on FcγRIIA and IIIB (19). In a neonatal mouse model of EBA, induced by the intradermal transfer of rabbit anti-mouse COL7 IgG, EBA development was driven by FcγRIII activation on neutrophils (20). In contrast, in an antibody-transfer model of EBA, activation FcγRIV on neutrophils was identified as a crucial step for disease induction (21). Absence of FcγRIIB expression was associated with a higher disease score (21, 22).

These findings suggest that activating FcγRs drive disease development during the effector phase using COL7-specific rabbit IgG and that FcγRIIB protects from disease development to some extent. Genetic association studies point toward a possible contribution of FcγRs to pemphigoid disease susceptibility. In humans, susceptibility to bullous pemphigoid is associated with a gain in *FCGR2C* and loss of *FCGR3B* gene copy numbers (23), as well as differences in the *FCGR3A* allotypes (24). In mice, blister formation in response to immunization with COL7 in an active model of EBA is linked to the H2s haplotype (25). Further, some EBA-resistant C57BL/6j (B6.j) mice developed disease symptoms when FcγRIIB-deficient mice on the B6.j background were repeatedly immunized with a COL7-GST fusion protein (26). Under these conditions, they found that around 50% of B6.j*-Fcgr2b*^−/−^ but none of the B6.j mice developed a clinical phenotype, while all mice developed aAbs which were deposited in the skin. Here, we aimed to determine whether FcγRIIB controls the development of autoimmune skin blistering disease in an active model of EBA. For this purpose, we crossed FcγRIIB-deficient mice to the EBA-susceptible inbred B6.s background (13, 27) and immunized B6.s and B6.s-*Fcgr2b*^−/−^ mice with the immunodominant von-Willebrand-factor-A-like domain 2 (vWFA2) region of COL7 (13). Then, we assessed the impact of FcγRIIB on the disease-permitting H2s haplotype on the formation of COL7-specific IgG aAbs, their epitope specificity, and functional properties, i.e., recruitment of neutrophils into the skin and their activation by activating FcγRs.

## Materials and Methods

### Mice

B6.SJL-H2s C3c/1CyJ (B6.s), C57Bl/6J (B6.j) and *Fcgr2b*^−/−^ on the B6.SJL (B6.s-*Fcgr2b*^−/−^) or C57Bl/6J (B6.j-*Fcgr2b*^−/−^) genetic background were bred and housed in a 12-h light-dark cycle at the animal facility of the University of Lübeck. Mice were killed by cervical dislocation and organs were harvested after intraperitoneal administration of a mixture of ketamine (100 μg/g) and xylazine (15 μg/g).

### Immunization-Induced EBA Model

Mice were immunized as previously described with some modifications (10, 26). Briefly, after anesthesia, B6.s wt and B6.s-*Fcgr2b*^−/−^ mice were immunized once with 60 μL of 1 mg/ml recombinant murine COL7 vWFA2 emulsified (1:1 mixture) in the non-ionic block copolymer adjuvant TiterMax^TM^ (ALEXIS Biochemicals, Norcross, GA) into each of the hind footpads. vWFA2 was produced after cloning of a commercially synthesized codon (Mr. Gene, Regensburg, Germany) optimized sequence in pTWIN (NEB, Frankfurt, Germany). In a second step, proteins were expressed in *E. coli* ER2566 and purified according to IMPACT™-TWIN protocol (NEB) as described (13). Subsequently, mice were evaluated for 6 weeks in 2-week intervals for the presence of skin lesions (i.e., erythema, blisters, erosions, crusts, and alopecia). Disease severity was expressed as percentage of body surface area affected by skin lesions. Serum samples were collected every second week. Serum, ear skin, and tail skin samples were obtained 2, 4, or 6 weeks after immunization and prepared for histopathologic examination and immunofluorescence (IF) microscopy. In accordance with animal welfare regulations, individual mice were sacrificed, if they lost more than 10% weight within 1 week and/or the affected skin surface reached >25% of the total skin surface area. Photographs were taken with standardized camera settings from multiple angles.

### Antibodies

For the antibody transfer-induced model, we used rabbit anti-vWFA2 antibodies (20 mg/mL) produced as described (28). For histology, we used AF647-labeled antibodies against IgG (Jackson Immuno Research; 25 μg/ml) or FITC-labeled antibodies against C3b (MP Biomedicals; 55500; 66 μg/mL). Further, we used polyclonal goat anti-mouse IgG directed against myeloperoxidase (MPO) (R&D Systems, AF3667, 0.2 mg/mL) and the monoclonal rat anti-mouse IgG antibody RB6-8C5 against Ly6G (Abcam, ab25377, 0.1 mg/mL). As secondary antibodies, we used goat anti rat IgG (H+L) labeled with Alexa Fluor 594 (Thermo Fisher Scientific, Invitrogen, A-11007, 2 mg/mL) or donkey anti goat IgG (H+L) labeled with Alexa Fluor 488 (Thermo Fisher Scientific, Invitrogen, A-11055, 2 mg/mL). Also, we used DAPI (4',6-Diamidino-2-Phenylindole, Dihydrochloride, Life Technologies; D3571, 5 mg/mL) for all immunofluorescence stainings. In addition, we used monoclonal rat anti-mouse IgG2a directed against CD19 labeled with Alexa Fluor 647 (Biolegend, 115522, 0.5 mg/mL) and monoclonal hamster anti-mouse IgG1 directed against CD3 labeled with FITC (BD, 553062, 0.5 mg/mL). For flow cytometric analysis, we used BV421-labeled antibodies against FcγRI (Biolegend; 139309; 0.2 mg/mL); FITC-labeled antibodies against FcγRIIB (provided by Falk Nimmerjahn; clone Ly17.2; 0.5 mg/mL), APC-labeled antibodies against FcγRIV (Biolegend; 149506; 0.2 mg/mL), APC-Cy7-labeled antibodies against Ly6G (Biolegend; 127623; 0.2 mg/mL) and secondary PE-labeled anti-goat antibodies (Santa Cruz biotechnology; sc-3857 0.4 mg/mL). Unlabeled antibodies against FcγRIII (Thermofisher; PA5-47230; 0.2 mg/mL); and against FcγRIV (Biolegend; 149506; 0.2 mg/mL) were used for blocking experiments.

### Reactive Oxygen Species (ROS) Release Assay

In this assay, intra- and extracellular ROS production from neutrophils were measured using luminol-amplified chemiluminescence after incubation with IC and controls, respectively. Neutrophils were isolated from bone marrow by flushing the femur and tibia with PBS using a 26G needle and red blood cells were removed with red blood cell lysis buffer (155 mM NH_4_Cl, 10 mM KHCO_3_, 0.1 mM EDTA at pH 7.2). Neutrophils were transferred to and kept in RPMI1640 (Genaxxon bioscience; C4116.0500) containing 1% fetal calf serum (PAA Cell Culture Company; A15-043) until use. For FcγR blocking, we incubated neutrophils with anti-FcγRIII (0.8 μg/mL) and/or anti-FcγRIV (8.5 μg/mL) for 1 h at 37°C, 5% CO_2_. Ninety-six-well microtiter plates (Greiner bio one; 655074) were coated with 20 μg/mL vWFA2 in PBS buffer. After washing with PBS-Tween (0.5%), sera from immunized B6.s wt mice were added in a 1:10 dilution for 1 h. After a second washing step neutrophils (2 × 10^6^/mL) with 5% of Luminol (Sigma Aldrich; 123072-5G) were added to each well. The luminescence, corresponding to the amount of liberated ROS in form of OH^−^, ${\text{O}}_{2}^{-}$, and H_2_O_2_ was measured for 1 h using a Fluostar Omega ELISA reader (BMG Labtech) at an absorbance rate up to 3600 and an average measurement interval of 1 s at 37°C.

### Determination of COL7-Specific IgG Serum Auto-Antibody Titers

Ninety-six-well plates were coated with vWFA2 (2.5 μg/ml) in 0.05M sodium-carbonate buffer for 10 min at room temperature. After blocking with 1% BSA-PBS, sera from immunized mice taken 2, 4, or 6 weeks after immunization were incubated on the plate for 1 h. The sera were diluted 1:10^3^, 1:10^4^, and 1:10^5^ in PBS. Then, HRP-coupled anti-mouse IgG (IgG1, IgG2b, IgG2c, or IgG-total; 1:6000 in PBS) were added for 1 h. Finally, 50 μL of Trimethoprim (TMP) was added and samples were measured on a Fluostar Omega (BMG Labtech) ELISA reader at 450 nm.

### vWFA2 Epitope Mapping of Sera From Immunized B6.s and B6.s-*Fcgr*2*b*^−/−^ Mice

Ninety-six-well plates were coated with vWFA2-derived polypeptides (2.5 μg/ml). The peptides were generated by peptides & elephants (Heringsdorf, German). Altogether, 19 20-mer peptides with 10 amino acid overlap (Table 1) were generated. They were dissolved in 0.05M sodium-carbonate buffer for epitope mapping for 10 min at room temperature. After blocking with 1% BSA-PBS, sera from immunized B6.s and B6.s-*Fcgr2b*^−/−^ mice were diluted 1:10^4^ and incubated for 1 h. Then, HRP anti-mouse was added for 1 h. Finally, 50 μL of TMB were added and samples were measured on a Fluostar Omega (BMG Labtech) at 450 nm.

**Table 1:** vWFA2-derived polypeptides used for epitope mapping.

**Name**	**Sequence**
Peptide 1	GRAMGACSH **GPVDVVFLLHA**
Peptide 2	**GPVDVVFLLHA** TRDNAHNAEA
Peptide 3	TRDNAHNAEA **VRRVLERLVS**
Peptide 4	**VRRVLERLVS** ALGPLGPQAA
Peptide 5	ALGPLGPQAA **QVGLLTYSHR**
Peptide 6	**QVGLLTYSHR** PSPLFPLNSS
Peptide 7	PSPLFPLNSS **HDLGIILRKI**
Peptide 8	**HDLGIILRKI** RDIPYVDPSG
Peptide 9	RDIPYVDPSG **NNLGTAVTTA**
Peptide 10	**NNLGTAVTTA** HRYLLASNAP
Peptide 11	HRYLLASNAP **GRRQQVPGVM**
Peptide 12	**GRRQQVPGVM** VLLVDEPLRG
Peptide 13	VLLVDEPLRG **DILSPIREAQ**
Peptide 14	**DILSPIREAQ** TSGLKVMALS
Peptide 15	TSGLKVMALS **LVGADPEQLR**
Peptide 16	**LVGADPEQLR** RLAPGTDPIQ
Peptide 17	RLAPGTDPIQ **NFFAVDNGPG**
Peptide 18	**NFFAVDNGPG** LDRAVSDLAV
Peptide 19	NGPG LDRAVSDLAV **ALCQAA**

*Sequences shown in bold highlight overlapping sequences*.

### IgG Auto-Antibody and C3b Deposition

Tissue-bound IgG aAb and C3b deposition was determined by direct IF microscopy of frozen sections in TissueTek (Sakura; Ref4583) using AF647-conjugated goat anti-mouse IgG (Jackson Immuno Research; 25 μg/mL), or FITC-conjugated goat anti-mouse C3b (MP Biomedicals; 55500; 66 μg/mL). Staining was evaluated using the Keyence BZ-9000E microscope with the BZII viewer and analyzer software. Intensity analysis was performed with the ImageJ comparison tools and results were compared by one-way ANOVA. First, we identified the basal membrane in bright field setting and then measured the intensity on the corresponding spot in the AF647 or FITC channel; subsequently we repeated this process for the epidermis as a negative control, where we found no specific IgG or C3 binding, and eventually calculated the difference between the measured intensities.

### Determination of FcγR Expression on Bone Marrow Neutrophils

To identify BM neutrophils, freshly harvested lower extremities were stored in PBS, flushed with PBS through 26G needles and red blood cell lysis was performed with lysis buffer (155 mM NH_4_CL; 10 mM KHCO_3_; 0.1 mM EDTA; pH = 7,22). First, we set a gate in the FSC SSC blot on lymphocytes excluding debris (**Figure 6A**). Within this gate, we identified neutrophils as Ly6G^+^ cells using APC-Cy7-labeled antibody against Ly6G (Biolegend; 127623; 0.7 μg/mL). To determine activating and inhibitory FcγR expression, the cell suspension was then stained with specific FcγRIIB (1.7 μg/mL), FcγRI, FcγRIII, FcγRIV (all 0.7 μg/mL) antibodies for 15 min at 4°C. Cells were washed and analyzed on the BD LSRII cell analyzer. Results were analyzed via FlowJoV10 (Tree Star, Ashland, OR).

### Assessment of MPO^+^, Ly6G^+^, CD3^+^, or CD19^+^ Cells in Mouse Ear Tissue

We identified MPO^+^ or Ly6G^+^ cells by direct IF microscopy of frozen ear sections in Tissue-Tek (Sakura, Ref4583) using polyconal goat IgG antibodies directed against mouse MPO (R&D Systems, AF3667) at a concentration of 2 μg/mL or monoclonal rat anti mouse Ly6G IgG (Abcam, ab25377, RB6-8C5) at a concentration of 1 μg/mL. Binding of the MPO or Ly6G-specific Abs was detected using either goat anti rat IgG (H+L) labeled with Alexa Fluor 594 (Thermo Fisher Scientific, Invitrogen, A-11007, 2 mg/mL) or donkey anti goat IgG (H+L) labeled with Alexa Fluor 488 (Thermo Fisher Scientific, Invitrogen, A-11055, 2 mg/mL) at a concentration of 4 μg/mL. To detect CD19^+^ B cells or CD3^+^ T cells via IF microscopy in frozen ear sections, we performed the following staining. Sections were stained with monoclonal rat anti-mouse IgG2a directed against CD19 labeled with Alexa Fluor 647 (Biolegend, 115522, 0,5 mg/mL) or monoclonal hamster anti-mouse IgG1 directed against CD3 labeled with FITC (BD, 553062, 0,5 mg/mL). Both antibodies were used at a concentration of 8,33 μg/mL. MPO and Ly6G stainings were evaluated using the EVOS M7000 imaging system (Thermo Fisher Scientific, Germany) and Celleste imaging analysis software (Thermo Fisher Scientific, Germany). CD3 staining was evaluated using the Olymus Fluor 1000 (Olympus, Germany) and FV10-ASW 4.2 (Olympus, Germany). Cell counting and fluorescence intensity analysis was performed with the ImageJ comparison tools. To enumerate MPO^+^ or Ly6G^+^ cells, we evaluated 10 pictures each with a size of 0.15 mm^2^ and 10 pictures for CD3^+^ and CD19^+^ with a size of 0.21 mm^2^ each.

### Statistical Analysis

Data were analyzed with GraphPad Prism Version 8.0. All data shown in the graphs are presented as mean values ± standard error of the mean (SEM). All groups analyzed for ROS release showed a normal distribution as tested by the Kolmogorov–Smirnov test. Two group comparison were done via unpaired Student's *t*-test or Mann Whitney *U*-test. Comparisons between multiple groups were done via non-parametric one-way ANOVA and Dunn's multiple comparisons as a *post-hoc* test. Differences between groups were considered significant when the *p-*value was < 0.05 (^*^p < 0.05, ^**^p < 0.01, ^***^p < 0.001).

## Results

### B6.s-*Fcgr*2*b*^−/−^ Mice Developed a Severe Clinical Phenotype Early After Immunization With COL7

**Figure 1:**
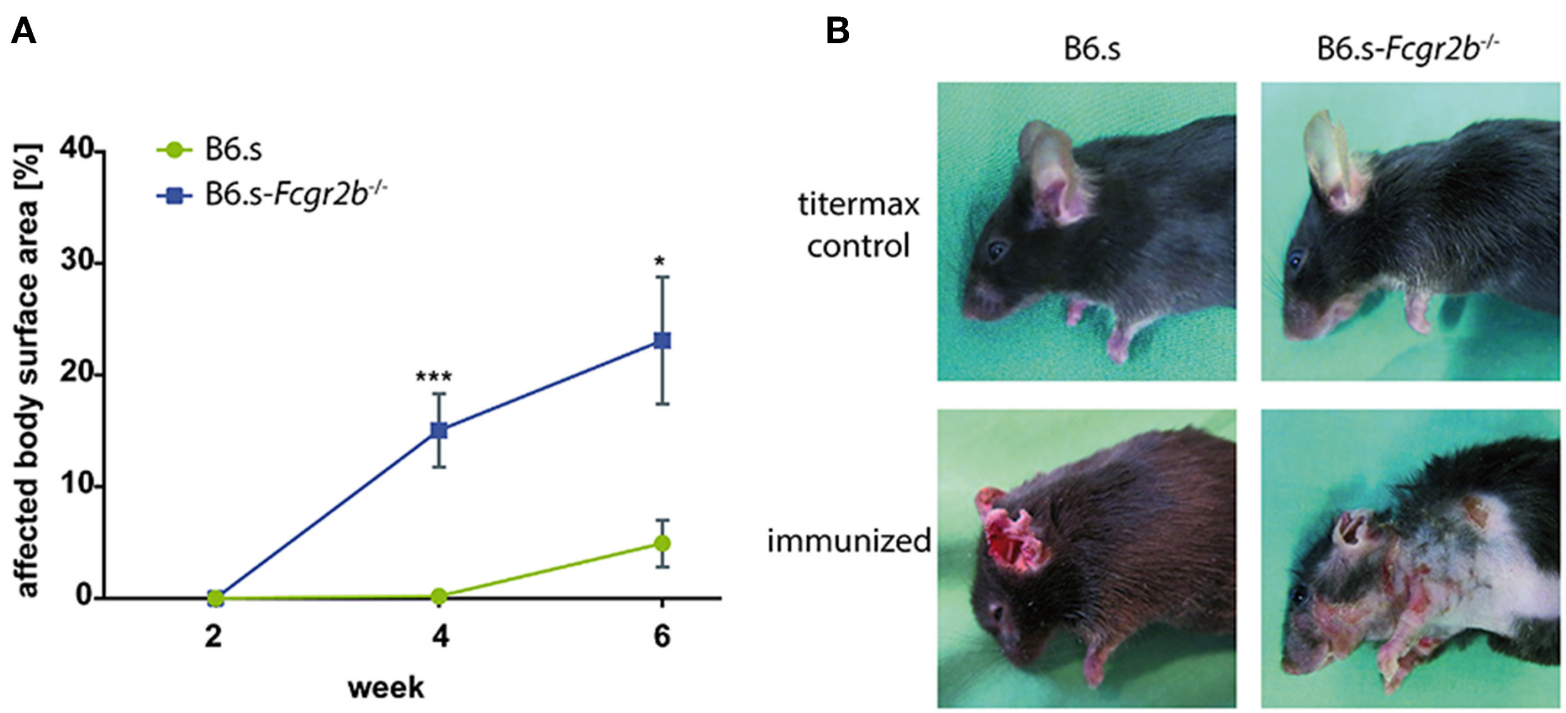
Disease development in B6.s-*Fcgr2b*^−/−^ mice compared to B6.s wt controls. vWFA2 was injected at a dose of 60 μg/mouse s.c. into each of the hind footpads of B6.s mice (*n* = 12) and B6.s-*Fcgr2b*^−/−^ mice (*n* = 12). Animals were monitored for 6 weeks. They developed erosions and crusts around the eyes, snout, ears, neck and torso. **(A)** Cumulative disease score of the affected body surface area between weeks 0 and 6 in B6.s-*Fcgr2b*^−/−^ mice as compared with B6.s wt controls. Statistical differences between wt and *Fcgr2b*^−/−^ mice were determined by an unpaired *t*-test. Values shown are the mean ± SEM; *n* = 12 for each group. (^*^p < 0.05; ^***^p < 0.001). **(B)** Representative pictures of non-immunized (upper row) and immunized (lower row, week 4) B6.s wt controls (left) and B6.s-*Fcgr2b*^−/−^ mice (right).

B6.s wt and B6.s-*Fcgr2b*^−/−^ were injected with vWFA2 together with Titermax to induce experimental EBA. Mice were scored as outlined in material and methods. We found that B6.s wt mice developed clinical symptoms between weeks 4 and 6 after vWFA2 immunization. In contrast, B6.s-*Fcgr2b*^−/−^ mice showed first clinical signs of skin blistering between weeks 2 and 4 after antigen administration. Importantly, the frequency of skin lesions was significantly higher in B6.s-*Fcgr2b*^−/−^ mice. The affected body surface area in wt mice was 4.9 ± 2.1% but 23.1 ± 5.7% in B6.s-*Fcgr2b*^−/−^ mice 6 weeks after immunization (Figure 1A). Already after 4 weeks, the affected body surface area reached 15.0 ± 3.3% in B6.s-*Fcgr2b*^−/−^ mice. At this time point, we observed almost no lesions in the skin of B6.s wt mice. Interestingly, lesions in wt mice at the early disease stage were limited to the ears and the head neck region, whereas in FcγRIIB-deficient mice extensive lesions occurred also on the torso and the upper limbs (Figure 1B). Due to the fulminant disease progression in mice lacking FcγRIIB, three mice needed to be sacrificed already 4 weeks after the initiation of the experiment. Such mice exceeded 25% of affected body surface area, which was set as the threshold to abort the experiment. None of the B6.s wt mice reached this criterion.

### The Production of vWFA2-Specific IgG Serum Auto-Antibodies Is Similar in B6.s and B6.s-*Fcgr*2*b*^−/−^ Mice

**Figure 2:**
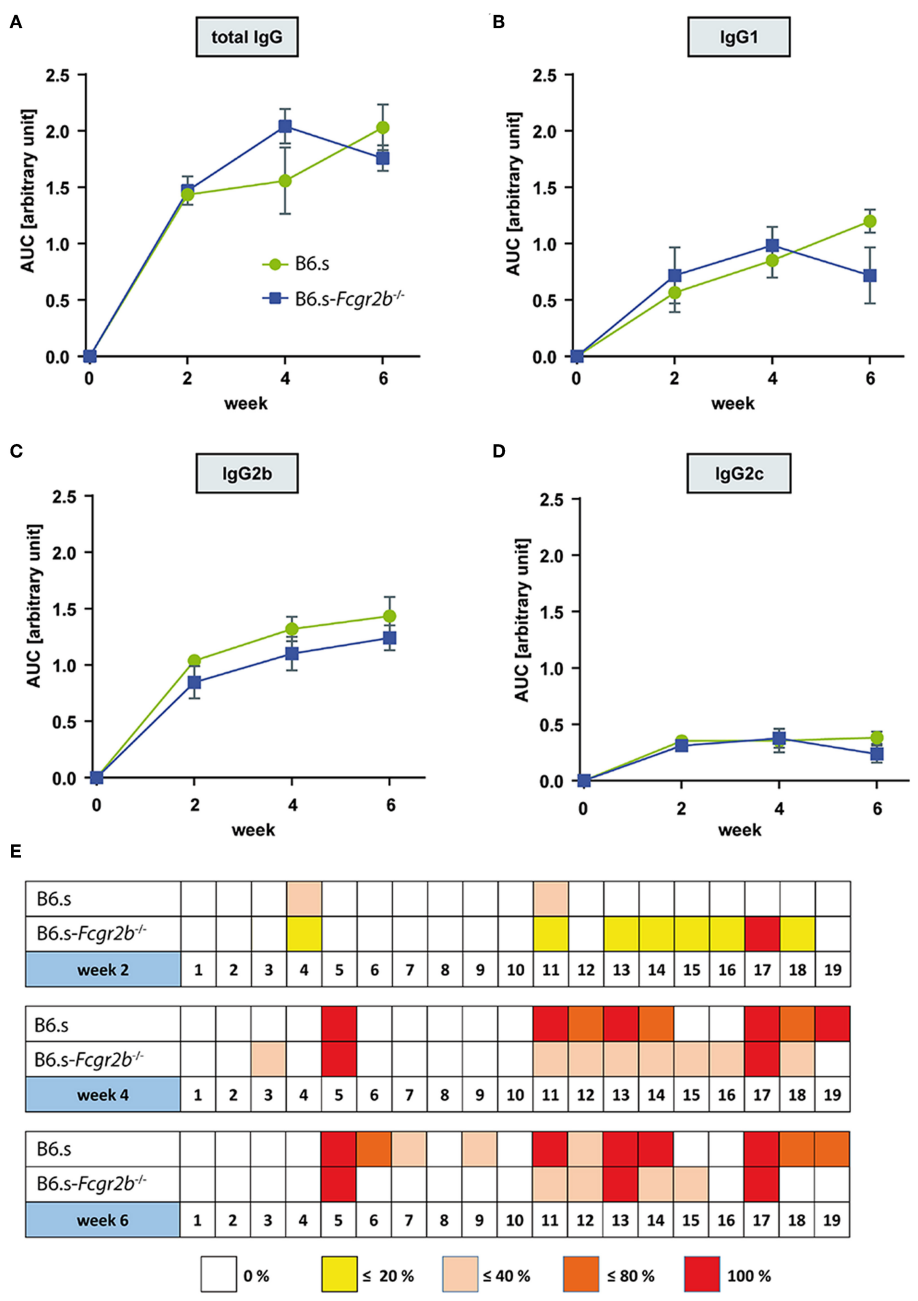
Comparison of vWFA2-specific serum IgG levels and epitope mapping during the first 6 weeks after vWFA2 immunization. Course of vWFA2-specific **(A)** total IgG, **(B)** IgG1, **(C)** IgG2b; and **(D)** IgG2c serum aAb levels. The IgG serum levels between B6.s wt and B6.s-*Fcgr2b*^−/−^ mice were similar. *n* = 4/group and time point. **(E)** Linear epitope mapping of vWFA2-specific total IgG using 20 amino acids long polypeptides of vWFA2 with an overlap of 10 amino acids. IgG peptide binding was determined via ELISA. *n* ≥ 3/group and time point. The depicted frequencies (0, <20, <40, <80, and 100%) relate to the percent of sera in B6.s wt or B6.s-*Fcgr2b*^−/−^ mice that reacted with respective peptide.

In search for mechanisms underlying the early disease development in mice lacking FcγRIIB, we first determined the vWFA2-specific serum IgG aAb titers in B6.s wt and B6.s-*Fcgr2b*^−/−^ mice 2, 4-, and 6-weeks post immunization. We analyzed the total IgG (Figure 2A) as well as the IgG1 (Figure 2B), IgG2b (Figure 2C) and IgG2c (Figure 2D) aAb subclasses. In both strains, we found significant amounts of anti-vWFA2 specific IgG1 and IgG2 aAbs 2 weeks after immunization, demonstrating that the generation of these aAbs starts during the first 2 weeks after immunization. Importantly, we observed no significant differences in the amount of IgG aAbs produced in mice lacking FcγRIIB when compared to B6.s wt mice (Figures 2A–D). Of note, the amount of vWFA2-specific IgG2c aAbs was lower than that of IgG1 or IgG2b aAbs in the two strains (Figure 2D). Taken together, the data show that the absence of FcγRIIB has no obvious impact on the quality and the quantity of IgG aAb production.

We next evaluated if differences in epitope specificity of the IgG aAb response could explain the observed clinical differences. For this purpose, we performed an epitope mapping with 19 overlapping 20-mer polypeptides spanning the entire NC1 domain of vWFA2 using the serum aAbs from B6.s wt and B6.s-*Fcgr2b*^−/−^ collected at weeks 2, 4, and 6. In week two, some sera from B6.s wt and B6.s-*Fcgr2b*^−/−^ mice both recognized an N-terminal peptide (peptide 4) and a peptide in the middle of vWFA2 (peptide 11). Interestingly, all sera from B6.s-*Fcgr2b*^−/−^ mice but none of the sera from B6.s wt mice recognized an epitope within the C-terminal peptide 17. Further, some sera from B6.s-*Fcgr2b*^−/−^ mice recognized epitopes in peptides adjacent to peptide 17, i.e., peptides 13–16 and peptide 18. Four weeks after immunization the number of epitopes recognized by the sera from B6.s wt mice increased markedly. Similar to the sera from B6.s-*Fcgr2b*^−/−^ mice, wt sera now recognized an epitope within peptide 17 and in the C-terminal part of vWFA2. Several of these epitopes overlapped with those recognized by the sera from FcγRIIB mice. Interestingly, sera from both mouse strains recognized an epitope within peptide 5. The epitope pattern recognized by the sera from B6.s-*Fcgr2b*^−/−^ mice was similar between weeks 4 and 6 with a dominant binding to C-terminal peptides and peptide 5 from the N-terminal part of vWFA2. Sera taken 6 weeks after immunization from B6.s wt mice showed a recognition pattern similar to that from B6.s-*Fcgr2b*^−/−^ mice, except that a few more epitopes within peptides from the N-terminal half of vWFA2 were recognized (peptides 6, 7, and 9) (Figure 2E). Taken together, our findings demonstrate that the early aAb response 2 weeks after immunization is more diverse in B6.s-*Fcgr2b*^−/−^ mice and already results in the formation of IgG aAbs that recognize epitopes within the C-terminal part of vWFA2. Importantly, several of these epitopes are also immunodominant in B6.s wt mice, although at a later time point (i.e., in weeks 4 and 6).

### IgG Auto-Antibody and C3b Deposition at the Dermal-Epidermal Junction Is Similar in B6.s and B6.s-*Fcgr*2*b*^−/−^ Mice

**Figure 3:**
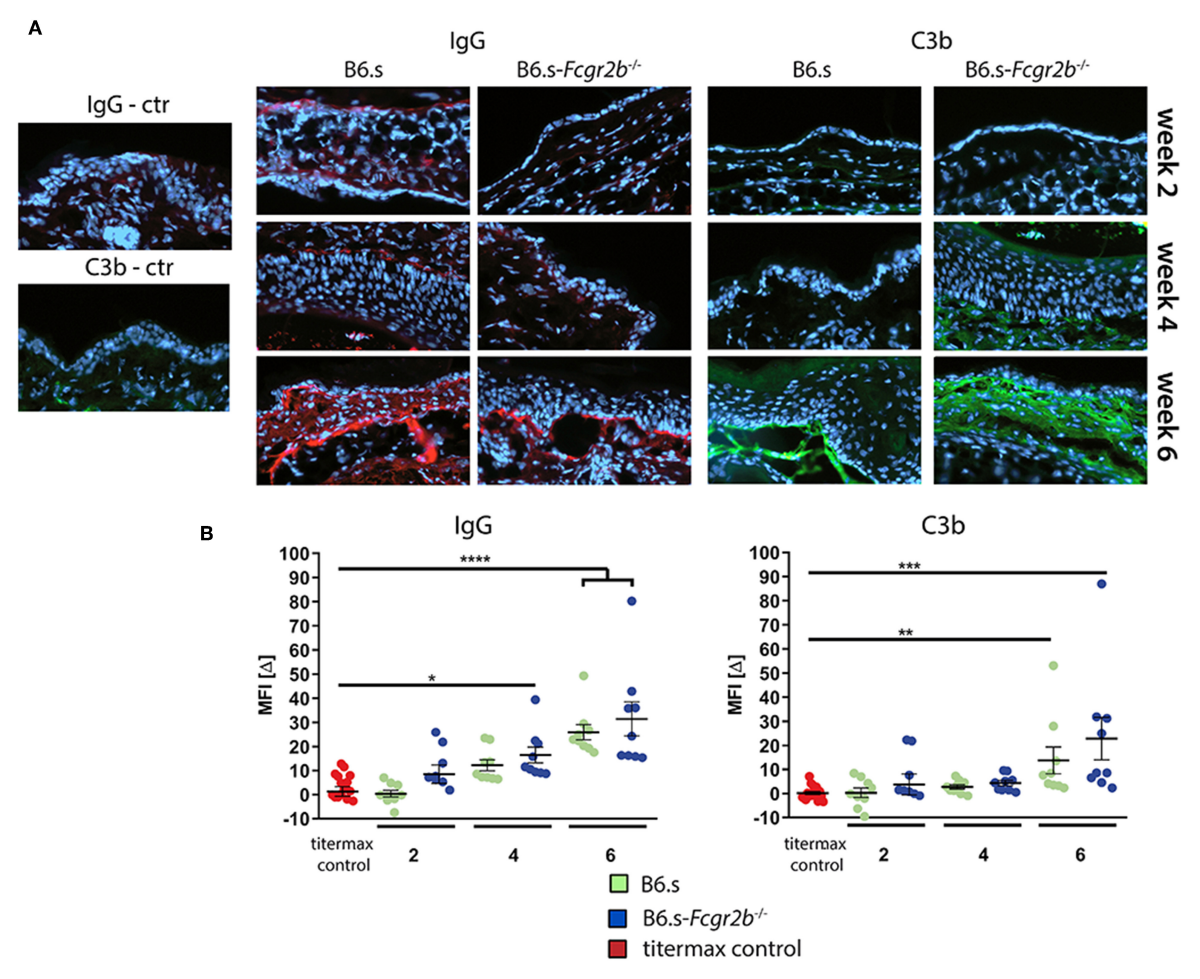
Direct immunofluorescence staining showing the deposition of IgG aAbs and C3b in perilesional biopsies of ear skin from B6.s wt and B6.s-*Fcgr2b*^−/−^ mice. **(A)** Linear deposits of murine IgG and C3b along the basal membrane zone. Representative immunofluorescence pictures of skin sections were taken at weeks 2, 4, and 6. Red = IgG aAb deposition; Green = C3b deposition. **(B)** Quantitative evaluation of IgG aAb and C3 deposition in B6.s wt and B6.s-*Fcgr2b*^−/−^ mice. Microscopic pictures were analyzed via ImageJ software. The staining intensity of the basal membrane zone was measured, and a reference area of the epidermis was subtracted from it defining the intensity value MFI[Δ] for each sample in three different high-power fields; Data are shown as scattered dot plot with mean and standard error of the mean. Differences between groups were analyzed by non-parametric one-way ANOVA with *post-hoc* Dunn's multiple comparisons test, (^*^p < 0.05, ^**^p < 0.01, ^***^p < 0.001, and ^****^p < 0.0001).

A prerequisite for the recruitment of effector cells to the skin that drive the formation of blisters, is the deposition of COL7-specific aAbs and the activation of complement at the (DEJ) zone. To assess, if the higher disease score seen in FcγRIIB deficient mice is associated with an increased IgG antibody or complement deposition at the DEJ, we stained skin samples taken 2, 4, or 6 weeks after vWFA2 immunization with IgG or C3b-specific antibodies. This was of particular interest, as our epitope mapping showed a more diverse epitope recognition using sera from B6.s-*Fcgr2b*^−/−^ mice (Figure 2B). As shown in Figures 3A,B, we found only minor IgG or C3b deposition 2 or 4 weeks after disease initiation in both strains. Disease-related IgG aAb and C3b deposition slightly increased 4 weeks after immunization in B6.s wt and B6.s-*Fcgr2b*^−/−^ mice (Figures 3A,B). However, we observed no significant differences between the two strains. Six weeks after antigen immunization, the deposition of IgG aAb and C3b increased markedly in both strains (Figure 3B), but still we observed no significant differences between the two strains. Collectively, our findings identified no major differences in IgG or C3b deposition during the first 6 weeks after disease initiation in B6.s wt or B6.s-*Fcgr2b*^−/−^ mice. These findings suggest that differences in IgG aAb or C3b deposition during the first 6 weeks after vWFA2 immunization do not account for the differences in disease development that we have observed between B6.s wt and B6.s-*Fcgr2b*^−/−^ mice. Surprisingly, the very high disease score that we found 6 weeks after immunization in B6.s-*Fcgr2b*^−/−^ mice seems to be independent of altered IgG aAb or C3b deposition at the DEJ.

### FcγRIIB-Deficient Mice Suffer From Strong Early Neutrophil Recruitment Into the Skin Associated With Strong Neutrophil Activation

**Figure 4:**
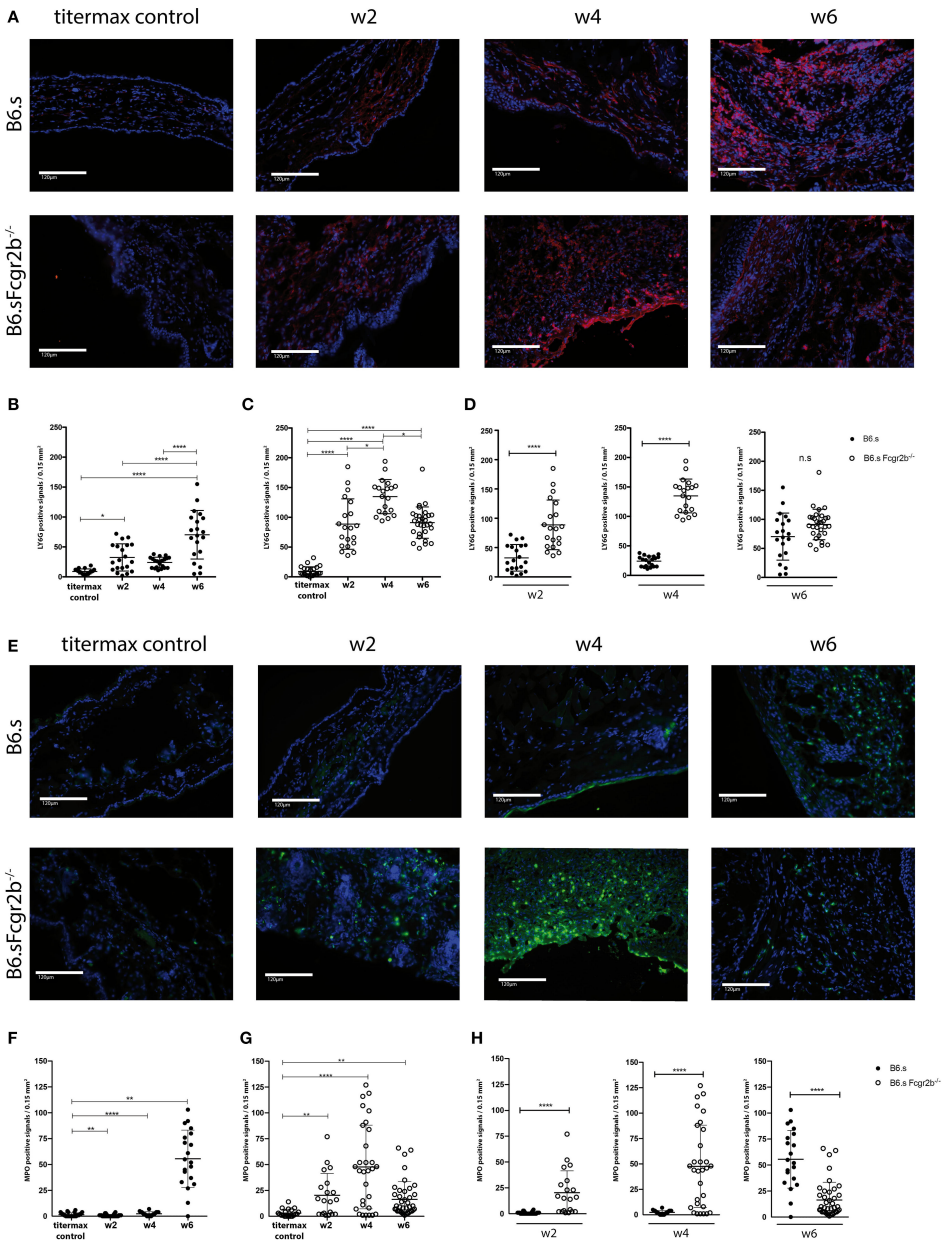
Strong early migration of Ly6G^+^ neutrophils to and MPO induction in the skin of B6.s-*Fcgr2b*^−/−^ but not B6.s wt mice. **(A)** Presence of Ly6G^+^ neutrophils in the skin of B6.s wt and B6.s-*Fcgr2b*^−/−^ mice. Representative immunofluorescence staining of ear sections taken 2, 4, and 6 weeks after immunization with vWAF2 antigen. Red = Ly6G^+^ neutrophils; Blue = DAPI. **(B,C)** Quantitative evaluation of Ly6G^+^ neutrophils in 0.15 mm^2^ sections of B6.s wt **(B)** and B6.s-*Fcgr2b*^−/−^ mice **(C)** during the 6 weeks observation period at week 2, 4, and 6. Titermax Control: representative staining of skin sections from B6.s wt and *Fcgr2b*^−/−^ mice treated with Titermax **(D)**. Comparison of the Ly6G^+^ neutrophil numbers in the skin of B6.s and B6.s-*Fcgr2b*^−/−^ mice at week 2 (left panel), 4 (middle panel), and 6 (right panel). Pictures were analyzed via ImageJ and Celleste software. **(E)** Presence of MPO^+^ signals in the skin of B6.s wt and B6.s-*Fcgr2b*^−/−^ mice. Representative immunofluorescence staining of ear sections taken 2, 4, and 6 weeks after immunization with vWAF2 antigen. Green = MPO^+^ signals; Blue = DAPI. **(F,G)** Quantitative evaluation of MPO^+^ signals in 0.15 mm^2^ sections of B6.s wt **(G)** and B6.s-*Fcgr2b*^−/−^ mice **(G)** during the 6 weeks observation period at week 2, 4, and 6. Control: Representative staining of skin sections from B6.s wt and *Fcgr2b*^−/−^ mice treated with Titermax **(D)**. Comparison of the MPO^+^ signals numbers in the skin of 6.s and B6.s-*Fcgr2b*^−/−^ mice at week 2 (left panel), 4 (middle panel), and 6 (right panel). Data in **(B–D)** and **(F–H)** are shown as scatter blot with mean ± SEM. Differences between week 2, 4, and 6 in **(B,C,F,G)** were analyzed by non-parametric one-way ANOVA. Differences between B6.s and B6.s-*Fcgr2b*^−/−^ mice **(D,H)** were evaluated by unpaired Student's *t*-test (^*^p < 0.05, ^**^p < 0.01, ^****^p < 0.0001).

Recruitment and activation of neutrophils to the DEJ is an important effector mechanism that drives blister formation in EBA (29). Therefore, we determined the number of Ly6G^+^ cells and the expression of MPO within the hole ear skin cut. First, we already observed the recruitment of Ly6G^+^ neutrophils into the skin of B6.s and B6.s-*Fcgr2b*^−/−^ mice 2 weeks after vWFA2 immunization (Figures 4A–D, Supplementary Figure 1). Importantly, the number of neutrophils was significantly higher in B6.s-*Fcgr2b*^−/−^ than in B6.s mice (32.5 ± 23 vs. 88.7 ± 42, Figure 4D, left panel). After 4 weeks of immunization the number of neutrophils further increased in the skin of B6.s-*Fcgr2b*^−/−^ mice but not in B6.s mice (24.1 ± 9 vs. 134.8 ± 29, Figure 4D, middle panel). After 6 weeks of immunization the number of neutrophils in the skin of B6.s-*Fcgr2b*^−/−^ mice somewhat decreased. Here, we need to take into account that 4–6 weeks after vWAF2 immunization, several B6.s-*Fcgr2b*^−/−^ mice had reached the termination criteria and had to be sacrificed. The number of neutrophils in B6.s wt mice increased 6 weeks after immunization as compared to the 4 week time point and reached numbers comparable to that of B6.s-*Fcgr2b*^−/−^ mice (70.3 ± 41 vs. 91.1 ± 26, Figure 4D, right panel).

**Figure 5:**
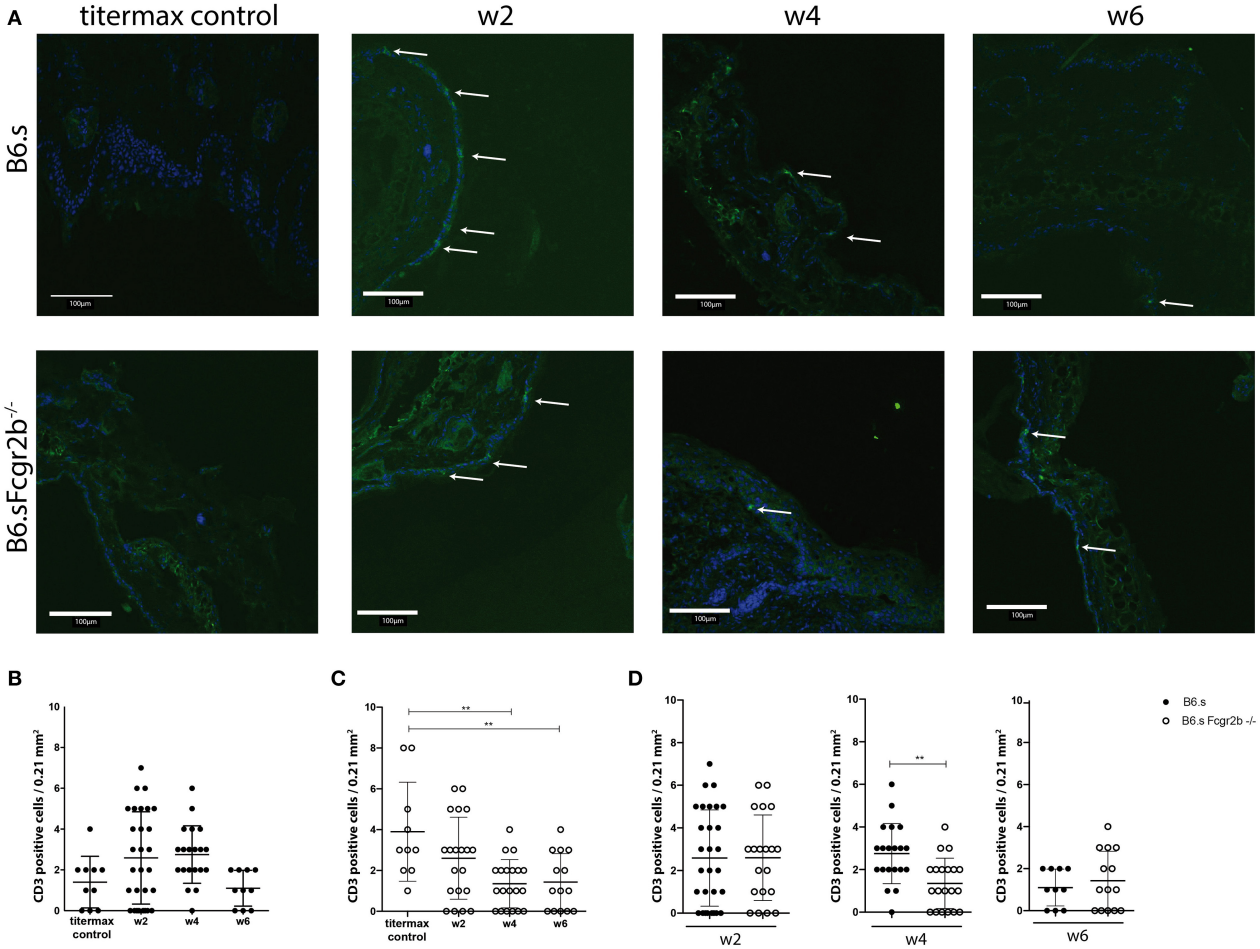
CD3^+^ numbers in the skin of B6.s wt and B6.s-*Fcgr2b*^−/−^ mice. **(A)** Presence of CD3^+^ T cells in the skin of B6.s wt and B6.s-*Fcgr2b*^−/−^ mice 2, 4, and 6 weeks after immunization with vWAF2 antigen. Pictures show immunofluorescence staining from ear sections (green = CD3^+^ T cells; blue = DAPI). Positive signals are indicated by arrows. **(B,C)** Quantitative evaluation of CD3^+^ T cells in 0.21 mm^2^ sections of B6.s wt **(B)** and B6.s-*Fcgr2b*^−/−^ mice **(C)** during the 6 weeks observation period at weeks 2, 4, and 6.^.^**(D)** Comparison of the CD3^+^ T cells numbers in skin of B6.s and B6.s-*Fcgr2b*^−/−^ mice at week 2 (left panel), 4 (middle panel), and 6 (right panel). Pictures were analyzed via ImageJ. Data in b-d are shown as scatter blot with mean ± SEM. Differences between week 2, 4, and 6 in **(B,C)** were analyzed by non-parametric one-way ANOVA test. Differences between B6.s and B6.s-*Fcgr2b*^−/−^ mice **(D)** were evaluated by unpaired Student's *t*-test (^**^p < 0.01).

Since not only the infiltration of the neutrophils into the skin, but also their activation plays a decisive role in disease development, we also determined the expression of MPO in the skin (Figures 4E–H, Supplementary Figure 1). Here, the difference in MPO expression between wild-type and Fc*y*RIIB-deficient animals was even more striking than that observed for neutrophil infiltration. We found strong MPO signals in B6.s-*Fcgr2b*^−/−^ but not in B6.s wt animals already in week 2 after immunization (Figure 4H). This signal increased toward week 4 in B6.s-*Fcgr2b*^−/−^, while it was still very low in B6.s wt mice. Only 6 weeks after immunization, we observed a strong MPO signal in B6.s wt mice, which was ever higher than that observed in B6.s-*Fcgr2b*^−/−^ mice. Again, the data in the B6.s-*Fcgr2b*^−/−^ mice are biased toward the phenotypically less affected animals. Together, these findings demonstrate a stronger and earlier recruitment of neutrophils into the skin of B6.s-*Fcgr2b*^−/−^ mice that were activated to produce high amounts of MPO when compared to B6.s wt mice. Next, we analyzed the infiltration of CD3^+^ cells into the skin, as previous publications have shown that T cells make an important contribution to the pathogenesis of EBA (30) (Figures 5A–D, Supplementary Figure 1). As compared with neutrophils, the infiltration with T cells was much lower. In B6.s wt mice we observed no significant differences between vWFA2-immunized and titermax-only treated mice (titermax: 1.4 ± 1, w2: 2.5 ± 2, w4: 2.7 ± 1.25, w6: 1.1 ± 0.8, Figure 5B). In B6.s-*Fcgr2b*^−/−^ mice, we noticed a decrease of CD3^+^ cells in the skin over time (titermax: 3.9 ± 2.3, w2: 2.6 ± 2, w4: 1.3 ± 1.1, w6: 1.4 ± 1.2, Figures 5C,D). In contrast to T cells, we did not find any B cells by direct IF in the skin cryosections. This is in accordance with previous studies, in which autoreactive B cells were almost exclusively found in the peripheral lymph nodes in the immunization-induced EBA mouse model (21, 31) (data not shown).

### Neutrophils From FcγRIIB- Deficient Mice Are More Sensitive to FcγRIII- and FcγRIV-Driven ROS Release by vWFA2-Specific IgG Auto-Antibodies

**Figure 6:**
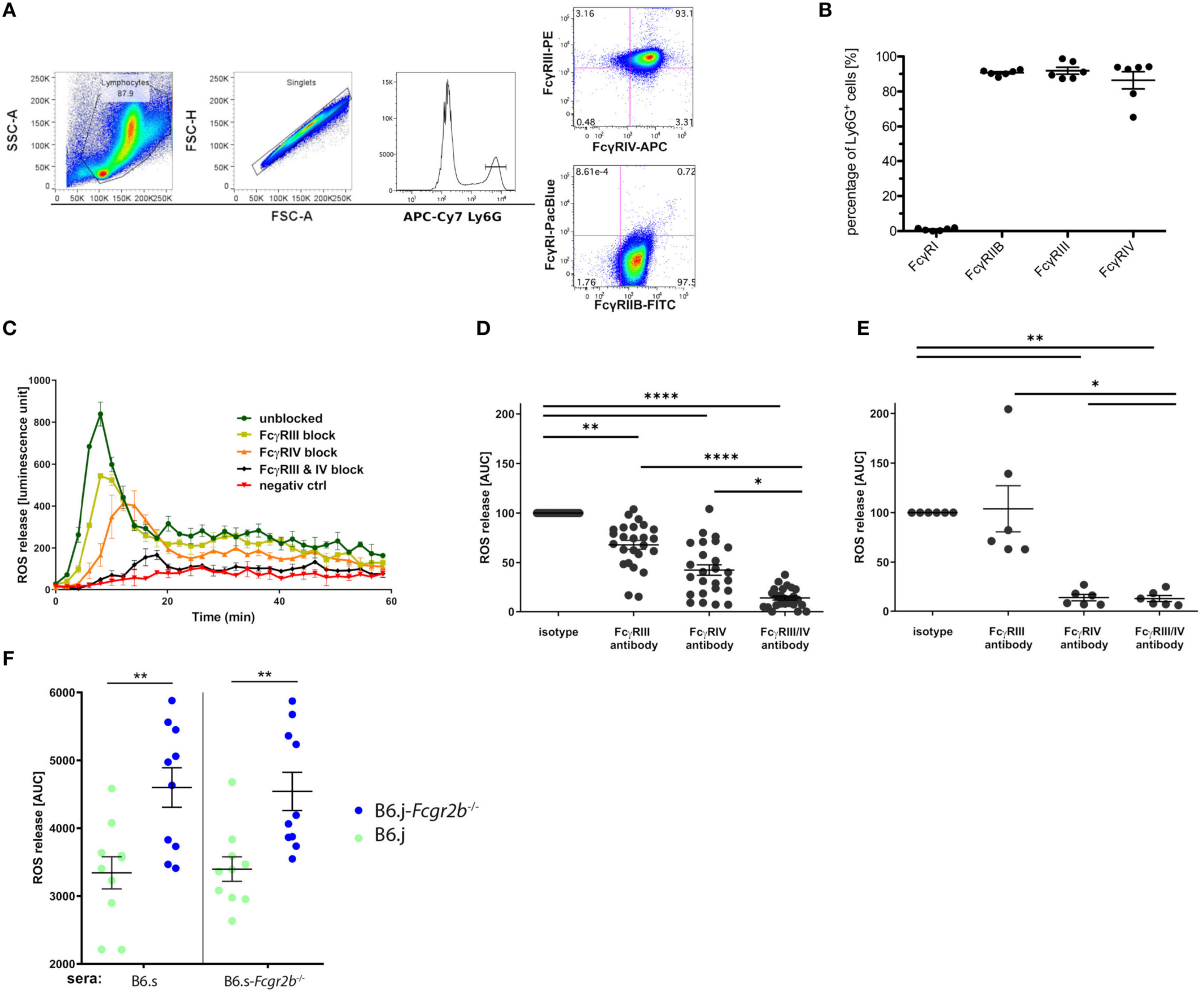
FcγR expression in bone marrow neutrophils and impact of vWFA2-specific IgG auto-antibodies on ROS release. **(A)** Flow cytometric analysis of FcγR expression in Ly6G^+^ bone marrow neutrophils. First, bone marrow cells were pregated using the FSC/SSC to exclude cell debris, residual erythrocytes, and lymphocytes, then singlets were excluded via FSC-A/FSC-H gating. In the last step, we gated on Ly6G^hi^ positive neutrophils, which were either stained for FcγRIII and FcγRIV expression (upper panel) or FcγRI and FcγRIIB expression (lower panel). **(B)** Quantitative assessment of FcγR expression in bone marrow neutrophils. High expression of FcγRIIB, FcγRIII, and FcγRIV (90.7 ± 1.4%; 98.4 ± 4.4%, and 86.43 ± 9.9%, respectively) but almost no FcγRI expression (0,96% ± 0,6%) in Ly6G^+^ neutrophils; *n* = 6/group, scatter blot with mean ± SEM. **(C)** ROS release from bone marrow neutrophils in response to sera from immunized wt B6.s mice (week 4) in the presence or absence of individual or combined FcγRIII/FcγRIV targeting. The graph is representative of 9 independent experiments. **(D)** Impact of FcγRIII and/or FcγRIV targeting on immune serum-driven ROS release from wt bone marrow neutrophils.; *n* = 25/group. Data are shown as scatter blot with mean ± SEM. The immune sera were from immunized wt B6.s mice (week 4). A non-parametric One-way ANOVA was used to determine significant differences and *post-hoc* Dunn's multiple comparison test to assess differences between individual groups (^*^p < 0.05; ^**^p < 0.001; ^****^p < 0.0001). **(E)** Impact of FcγRIII and/or FcγRIV targeting on immune serum-driven ROS release from wt bone marrow neutrophils. The immune sera were taken from rabbits immunized with vWFA2, *n* = 6/group. Data shown are scattered dot blot with mean ± SEM (^*^p < 0.05; ^**^p < 0.01) **(F)** Potency of sera from B6.s and B6.s-*Fcgr2b*^−/−^ mice to induce ROS release from B6.s wt (green) or B6.s-*Fcgr2b*^−/−^ (blue) neutrophils. Neutrophils from B6.j-*Fcgr2b*^−/−^ mice produced more ROS than those from wt B6.s mice in response to sera from either B6.s or B6.s-*Fcgr2b*^−/−^ mice. *n* = 10 / group. Data shown are scattered dot blot with mean ± SEM. Unpaired Mann–Whitney test was used to determine significant differences between groups (^**^p < 0.01).

The most important effector function of neutrophils in the skin is the release of ROS through aggregation of activating FcγRs (22). Thus, we tested the potential of the sera from immunized wt and B6.s-*Fcgr2b*^−/−^ mice to induce ROS release. For this purpose, we used bone marrow neutrophils. In a first step, we determined the expression of activating FcγRs and the inhibitory FcγRIIB. We found that Ly6G^+^ bone marrow neutrophils are expressing the activating FcγRs FcγRIII (98.4%) and FcγRIV (86%), whereas FcγRI is barely detectable. Similar to FcγRIII and FcγRIV, FcγRIIB was strongly expressed on 90% of all bone marrow neutrophils (Figures 6A,B). In the antibody transfer model of EBA, an exclusive role for FcγRIV activation has been shown regarding the induction of ROS release by COL7-specific rabbit IgG (21). In mice, FcγRIV activation is largely independent from FcγRIIB counterbalance (32). In contrast, FcγRIII activation by IgG1 antibodies heavily depends on FcγRIIB expression, as IgG1 antibodies bind to FcγRIIB and FcγRIII with similar affinity (1).

In the second step, we thus aimed to determine, which FcγRs are activated by the sera from immunized B6.s wt and B6.s-*Fcgr2b*^−/−^ mice to drive ROS release from bone marrow neutrophils. For this purpose, we stimulated bone marrow neutrophils with sera from wt mice. As shown in Figure 6C, wt sera induced a strong peak within the first 10 min after administration. Thereafter, ROS production steadily declined during the next 50 min. FcγRIII blockade did not change the kinetic of the ROS release. However, the total amount of generated ROS decreased to 67.9 ± 4.5% of that induced in the presence of the isotype control. In contrast, the ROS release was delayed when FcγRIV was targeted. Further, the amount of ROS released from the bone marrow neutrophils decreased to 42.4 ± 5.3% of that induced by the B6.s wt serum in the presence of the isotype control. The joint blocking of FcγRIII and FcγRIV abrogated the wt serum-induced ROS release from bone marrow neutrophils. In fact, the amount of ROS production (13.8 ± 2.0%) reached the level of unstimulated neutrophils (Figure 6D and data not shown). In a similar experimental setup, we tested the potential of rabbit anti-mouse COL7-antibodies to induce ROS in the presence of absence of blocking antibodies against FcγRIII and/or FcγRIV. Confirming previous findings (21), we found that vWFA2-specific rabbit IgG, that are used in antibody-transfer model of EBA, drive ROS release through exclusive activation of FcγRIV, whereas FcγRIII does not seem to play any role (Figure 6E).

Finally, we compared the ROS-inducing potential of anti-vWFA2 IgG aAbs from B6.s wt or B6.s-*Fcgr2b*^−/−^ mice using bone marrow neutrophils from either B6.j wt or B6.j-*Fcgr2b*^−/−^ mice. We found a markedly and significantly increased ROS production induced by sera from either B6.s wt or B6.s-FcγRIIB-deficient mice, when we used bone marrow neutrophils from B6.j-*Fcgr2b*^−/−^ mice instead of neutrophils from B6.j wt mice (Figure 6F). These findings demonstrate a critical role for FcγRIIB in controlling IgG aAb -driven ROS release from neutrophils suggesting that the main effect underlying the increased phenotype that we observed in B6.s-*Fcgr2b*^−/−^ mice results from insufficient control of FcγRIIB-deficient neutrophil activation by IgG aAbs through FcγRIII activation.

## Discussion

EBA is a rare autoimmune skin disease, with an incidence of 0.08–0.5 new cases per million per year, in which autoantibodies to COL7 bind to the DEJ and induce blister formation on the skin and erosions on the mucus membranes (33–35). Such aAbs against COL7 are detectable in the basement membrane and in the serum. Despite advancements in the understanding of the pathogenesis, effective treatment still remains a challenge (35). Experimental models of EBA support the idea that binding of aAbs to COL7 mediates inflammation and triggers blister formation (18, 36). In an antibody-transfer model (passive model) of EBA, using rabbit-derived antibodies against COL7, it was shown that these antibodies activate the complement system, resulting in the release of pro-inflammatory cytokines and chemokines, and neutrophil recruitment into the skin (18). In this model neutrophils interact with the rabbit anti-mouse COL7 IC exclusively via FcγRIV, resulting in the release of ROS and proteolytic enzymes (18, 21, 37). The different subclasses of IgGs differ in their ability to bind to distinct FcγRs and their potency activate them. Further, the relative distribution of activating FcγRs and the inhibitory FcγR, FcγRIIB, determines the magnitude of IgG-driven immune cell activation. Despite their undisputed beneficial role in translating antibody-responses into cellular responses, they are also involved in the pathogenesis of various autoimmune bullous diseases. Clearly, FcγRs play an essential role in the pathogenesis of EBA (38). The main goal of this study was to assess whether the inhibitory FcγR, FcγRIIB, controls the development of autoimmune skin blistering disease in the active model of EBA, in which we immunized mice with the immunodominant vWFA2 region of COL7.

To the best of our knowledge, this is the first report on the role of FcγRIIB in an active model of EBA, in which congenic FcγRIIB-deficient and wt mice with the same disease-permitting H2s haplotype (B6.s background) have been compared side-by-side regarding the impact of FcγRIIB on the B and the effector cell responses. Sitaru et al. used FcγRIIB-deficient mice on the EBA-resistant C57BL/j (B6.j) background (H2b haplotype) and immunized the mice repeatedly (4 x) with GST-tagged COL7. Under these conditions, they found that around 50% of B6.j-*Fcgr2b*^−/−^ but none of the B6.j mice developed a clinical phenotype, while all mice developed aAbs which were deposited in the skin (26). Importantly, GST-peptide tag coupled to the murine fibronectin III–like domains (FNIII) 7 and 8 of COL7 (termed mCOL7C) modulates the mCOL7C-specific plasma cell response and the induction of EBA (13, 31). The immunization scheme that we used in our study drives disease development independent from immune responses to other antigens, i.e., GST. Also, the vWFA2 domain of COL7 is an immunodominant epitope in humans (13, 39), which is why we used this part of the COL7 protein without GST-tag for immunization. Thus, the active EBA model used in this study is based on a more specific immune response to a more EBA-relevant protein fragment of COL7.

In a first step, we analyzed the role of the inhibitory FcγRIIB in controlling the disease and its impact on aAb formation, since this is the only FcγR expressed on B lymphocytes (40). We found that B6.s-*Fcgr2b*^−/−^ mice suffer from earlier disease onset and faster disease progression resulting in more severe disease phenotype than their B6.s wt counterparts. Interestingly, B6.s wt mice show first signs of the disease between week 4 and 6, whereas FcγRIIB-deficient mice were already affected between week 2 and 4 after immunization. Even more striking was the fact that all wt mice reached the endpoint of the experiment after 6 weeks, while some of the FcγRIIB-deficient mice had to be sacrificed, as they reached the pre-defined abortion criterium of more than 25% affected body-surface area. This finding of an important contribution of FcγRIIB to disease onset and severity is in agreement with previous reports that have linked polymorphisms in FcγRIIB with autoimmune diseases including SLE (41, 42), rheumatoid arthritis (43), anti-GBM diseases (44), IC-mediated alveolitis (45), and collagen-induced arthritis (46).

To further delineate the role of FcγRIIB in EBA development, we characterized the antibody response in B6.s wt and B6.s-*Fcgr2b*^−/−^ mice during the course of the disease to determine whether the lack of FcγRIIB would affect the vWFA2-specific IgG response. In the past, it was shown that polymorphisms in the promoter and regulatory region of *FCGRIIB* are associated with a reduced expression of this receptor, which caused a higher susceptibility for autoimmune diseases (47–49) suggesting an important role for FcγRIIB in controlling B cell activity to prevent autoimmune responses. This was confirmed by a study using B cell-specific conditional FcγRIIB-deficient mice, in which the absence of FcγRIIB on B cells increased the humoral response after T-dependent immunization and aggravated disease severity in chicken collagen-induced arthritis (50). In contrast, the humoral immune response decreased in the same arthritis model, when mice were used that overexpress FcγRIIB on B cells (51). However, Yilmaz-Elis et al. (52) were not able to reproduce the increased levels of aAbs. In light of these findings, we determined in the present study the aAb response in B6.s wt and B6.s-*Fcgr2b*^−/−^ mice during the course of EBA. To our surprise, we found no differences in the levels of vWFA2-specific IgG aAbs during the course of the disease. B6.s wt and B6.s-*Fcgr2b*^−/−^ mice showed the same kinetic and amount of IgG aAb production, suggesting that the early disease onset and the severe clinical phenotype in B6.s-*Fcgr2b*^−/−^ mice were caused by other mechanisms.

During an autoimmune response, tissue damage can lead to the priming of self-reactive T or B cells, regardless of the initial insult resulting in a phenomenon known as epitope spreading. Thus, we tested whether the severe lesions with massive tissue destruction of the skin observed in FcγRIIB-deficient mice would lead to strong epitope spreading and/or IgG aAbs produced in FcγRIIB-deficient mice would recognize epitopes different from those recognized by IgG aAbs from B6.s wt mice as a measure that might account for the aggravated phenotype in B6.s-*Fcgr*2*b*^−/−^ mice. In epitope-mapping experiments, we observed several immunodominant epitopes that were recognized by IgG aAbs from B6.s wt and FcγRIIB deficient sera [i.e., (5, 13, 17)]. However, B6.s-*Fcgr2b*^−/−^ mice showed an accelerated target evolution. All sera from B6.s-*Fcgr2b*^−/−^ mice but none from B6.s wt mice recognized the immunodominant epitope in peptide 17 already 2 weeks after vWFA2 immunization. Further, <20% of FcγRIIB- but none of the B6.s wt sera reacted with the immunodominant epitope in peptide 13 at this time point. Also <20% of the B6.s-*Fcgr2b*^−/−^ sera already recognized several peptides that overlapped with peptides 13 (i.e., peptide 14) or 17 (i.e., peptides 16 and 18) in this early stage of disease development, while B6.s wt mice reached the highest diversity in their aAb response only in week 6 (11 targets, 40–100%). These findings suggest a direct effect of FcγRIIB on B cells for antibody profiling or an indirect effect through increased antigen presentation on APCs such as skin DCs, eventually resulting in a more severe inflammation at the DEJ. Clearly, the early disease onset with a higher magnitude of inflammation in the absence of FcγRIIB is associated with strong antibody spreading before disease onset.

Interestingly, we found almost no (week 2) or only weak (week 4) IgG aAb deposition and consecutive complement deposition in the early stage of the disease in both mouse strains although IgG aAb serum titers were already high 2 weeks after immunization. However, 6 weeks after immunization we found markedly increased levels of IgG aAb and complement deposition at the DEJ, but—as for the IgG serum titers—there was no detectable difference between wt and B6.s-*Fcgr2b*^−/−^ mice. Based on these findings we concluded that the massive clinical phenotype that we observed in FcγRIIB-deficient mice was not caused by an aggravated IgG aAb or C3b deposition, since there was no detectable difference compared to the wt mice.

Next, we investigated whether the absence of FcγRIIB affects the recruitment and activity of neutrophil granulocytes into the skin. Interestingly, we already found strong neutrophil migration into the skin of B6.s-*Fcgr2b*^−/−^ mice 2 weeks after immunization, when aAb deposition at the DEJ was low. At week 4, the number of neutrophils was even higher and exceeded by far the number of neutrophils in B6.s wt mice. In week 6, neutrophil skin infiltration was high in B6.s wt and B6.s-*Fcgr2b*^−/−^ mice. This observation matches the strong disease phenotype in B6.s-*Fcgr2b*^−/−^ that we had observed in week 4 and the delayed disease onset in B6.s wt mice at week 6. This view was supported by the MPO expression as an indicator of neutrophil activity. The strong MPO expression in B6.s-*Fcgr2b*^−/−^ mice in weeks 2 and 4 paralleled the high number of number of neutrophils. Even more striking, we found at best minor MPO expression in the skin of B6.s wt mice suggesting minor activation of the recruited neutrophils. These findings suggest that FcγRIIB activation controls the recruitment and the activation of neutrophils into the skin. In future studies, it will be important to delineate the mechanisms underlying the FcγRIIB-mediated regulation of neutrophil homing into the skin and their activation in the skin tissue. Clearly, FcγRIIB expression controls FcγRIII-driven activation of neutrophils. Interestingly, in the antibody-transfer model of EBA, in which COL7-specific Abs from rabbits are injected into mice, the activation of neutrophils and the development of skin lesions is exclusively mediated through FcγRIV (21). Based on our findings that vWFA2-immunized B6.s wt and B6.s-*Fcgr2b*^−/−^ mice produced IgG1, IgG2b and IgG2 aAbs, which were deposited at the DEJ and resulted in distinct recruitment and neutrophil activation, we aimed to determine the individual contribution of each IgG isotype and the corresponding FcγR to disease development. For this purpose, we conducted *in vitro* experiments, in which we induced ROS release from bone marrow neutrophils using sera from B6.s wt mice. The B6.j wt neutrophils that we used as ROS producers were treated with antibodies to block either FcγRIII, FcγRIV individually or in combination. In these experiments, we showed that the blockade of FcγRIV was not sufficient to completely block the ROS release by bone marrow neutrophils. In contrast to the findings with rabbit anti-mouse COL7 Abs, the sole blockade of FcγRIII was almost as efficient in reducing ROS release from neutrophils as FcγRIV targeting. Importantly, only the combined targeting of FcγRIII and FcγRIV reduced ROS release to the baseline level. This finding is of major importance, as it demonstrates that not only IgG2b/c but also IgG1 aAbs can drive neutrophil activation in the active EBA model. Obviously, the passive EBA model using rabbit IgG Abs is biased toward FcγRIV-mediated neutrophil activation due to the fact that rabbit IgG seems to exclusively bind to FcγRIV in the murine systems. Our findings demonstrate that IgG aAbs raised in response to vWFA2 are heterogenous and comprise Abs of the IgG1 and the IgG2 subtype, both of which drive neutrophil activation. Importantly, IgG1-mediated FcγRIII activation is controlled by FcγRIIB co-expression, whereas IgG2c-driven neutrophil activation by FcγRIV is largely independent of FcγRIIB co-expression (53). We found that stimulation of bone marrow neutrophils from FcγRIIB-deficient mice with sera from either B6.s wt or B6s.FcγRIIB-deficient mice immunized with vWFA2 resulted in a markedly higher ROS release than stimulation of bone marrow neutrophils from FcγRIIB-sufficient wt mice. These data confirm the importance of FcγRIIB as an important negative regulator of IgG aAb-driven ROS release from neutrophils in active experimental EBA, most likely induced through IgG1 aAbs that trigger FcγRIII. Future studies that aim to determine the immune mechanisms underlying EBA development should take this difference into account.

The current standard EBA treatment comprising corticosteroids, rituximab, intravenous immunoglobulins (IVIG), colchicine and other broadly immune suppressive drugs is associated with several severe side effects. Clearly, an unmet need exists for more specific therapies. Our data suggest that controlling FcγRIIB or targeting FcγRIII and FcγRIV might serve as a novel treatment strategy in EBA patients.

In summary, we found that FcγRIIB is crucially involved in the development of skin blistering in an immunization-induced mouse model of EBA. It results in an early disease onset and the development of a very severe disease phenotype through regulation of the immune response at several levels. First, FcγRIIB suppresses early epitope spreading and strong production of IgG aAbs that recognize an immunodominant epitope within the C-terminus of the NC-1 domain of vWFA2. Secondly, FcγRIIB protects from strong early neutrophil infiltration to and activation of neutrophil granulocytes in the skin. Thirdly, FcγRIIB is critical to counterbalance FcγRIII activation by IgG1 aAbs which drive enhanced ROS release from neutrophils leading to tissue destruction at the DEJ. Our findings that FcγRIII and FcγRIV drive disease development in active EBA should be considered in future studies that aim to delineate the immune mechanisms underlying EBA development.

## Data Availability Statement

All datasets generated for this study are included in the article/Supplementary Material.

## Ethics Statement

The animal study was reviewed and approved by animal Care and Use Committee of Schleswig-Holstein in the Ministerium für Energiewende, Landwirtschaft, Umwelt, Natur und Digitalisierung Schleswig-Holstein (V242-22797/2016(43-4/16).

## Author Contributions

JK, CK, JT, BK, L-CF, CS, KB, RL, and FN contributed to the study design, performance, and manuscript draft. BK, JT, and L-CF analyzed the experimental data. BK, JT, JK, and CK wrote and revised the manuscript.

### Conflict of Interest

The authors declare that the research was conducted in the absence of any commercial or financial relationships that could be construed as a potential conflict of interest.

## Supplementary Material

Supplementary Figure 1 Isotype Control staining of cryosections from B6.s mice. Representative pictures of direct IF staining taken 6 weeks after vWFA2 immunization from B6.s mice. Shown is the direct comparison of IgG Ab isotype control staining (left) vs. IgG antigen-specific staining (right) for **(A)** Ly6G; **(B)** MPO; and **(C)** CD3.
