# Supplementary figures and images for: Fcγ Receptor IIB Controls Skin Inflammation in an Active Model of Epidermolysis Bullosa Acquisita
Source: Front Immunol. 2020 Jan 14;10:3012. doi: 10.3389/fimmu.2019.03012 (PMC6971089; doi:10.3389/fimmu.2019.03012)

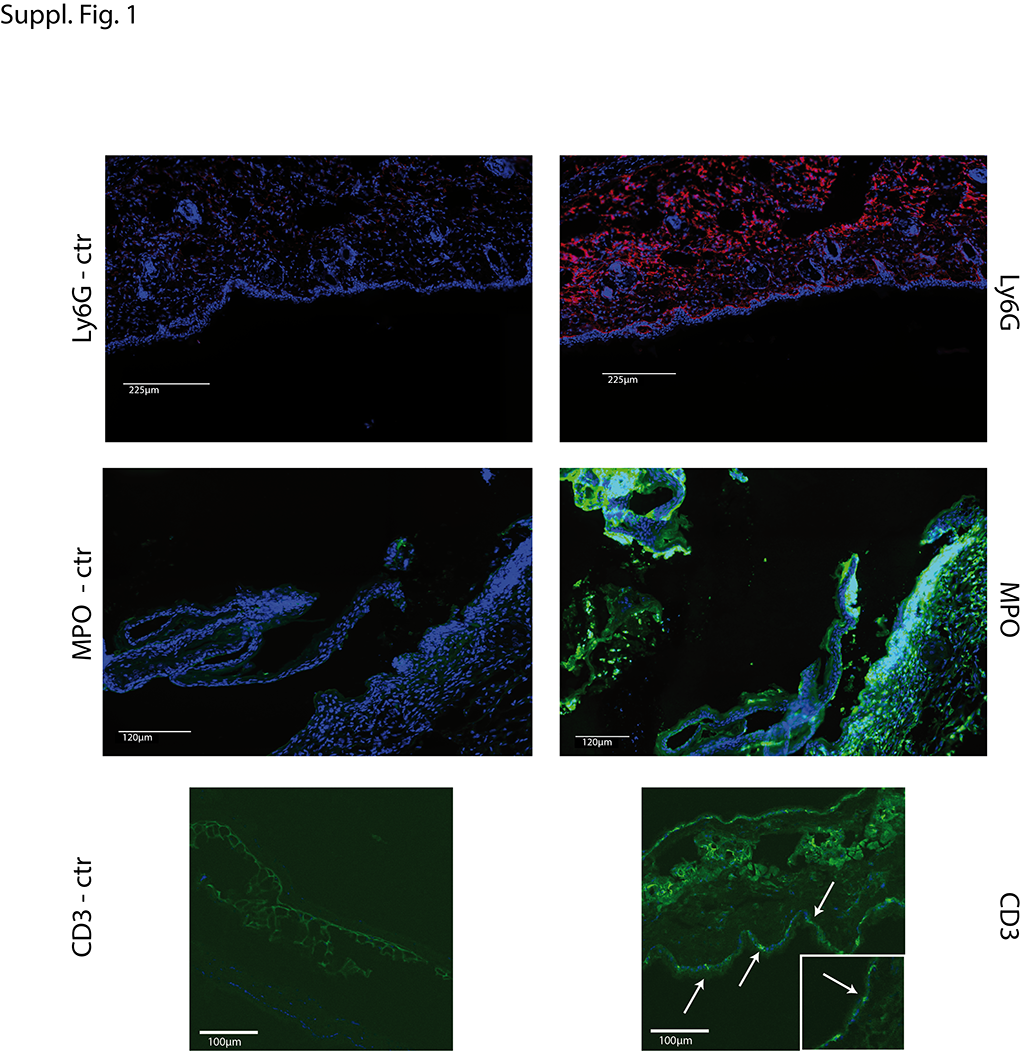

Supplement: Supplementary Figure 1 — Isotype Control staining of cryosections from B6.s mice. Representative pictures of direct IF staining taken 6 weeks after vWFA2 immunization from B6.s mice. Shown is the direct comparison of IgG Ab isotype control staining (left) vs. IgG antigen-specific staining (right) for (A) Ly6G; (B) MPO; and (C) CD3. [file Image_1.TIF]
